# A precise gene delivery approach for human induced pluripotent stem cells using Cas9 RNP complex and recombinant AAV6 donor vectors

**DOI:** 10.1371/journal.pone.0270963

**Published:** 2022-07-07

**Authors:** Koollawat Chupradit, Nontaphat Thongsin, Chatchai Tayapiwatana, Methichit Wattanapanitch

**Affiliations:** 1 Research Department, Siriraj Center for Regenerative Medicine, Faculty of Medicine Siriraj Hospital, Mahidol University, Bangkok, Thailand; 2 Center of Biomolecular Therapy and Diagnostic, Faculty of Associated Medical Sciences, Chiang Mai University, Chiang Mai, Thailand; 3 Department of Immunology, Faculty of Medicine Siriraj Hospital, Mahidol University, Bangkok, Thailand; 4 Division of Clinical Immunology, Department of Medical Technology, Faculty of Associated Medical Sciences, Chiang Mai University, Chiang Mai, Thailand; 5 Center of Innovative Immunodiagnostic Development, Faculty of Associated Medical Sciences, Chiang Mai University, Chiang Mai, Thailand; Purdue University, UNITED STATES

## Abstract

Genome editing in human induced pluripotent stem cells (hiPSCs) offers a potential tool for studying gene functions in disease models and correcting genetic mutations for cell-based therapy. Precise transgene insertion in hiPSCs represents a significant challenge. In the past decade, viral transduction has been widely used due to its high transduction efficiency; however, it can result in random transgene integration and variable transgene copy numbers. Non-viral-based strategies are generally safer but limited by their low transfection efficiency in hiPSCs. Recently, genome engineering using adeno-associated virus (AAV) vectors has emerged as a promising gene delivery approach due to AAVs’ low immunogenicity, toxicity, and ability to infect a broad range of cells. The following protocol describes the workflow for genome editing in hiPSCs using the CRISPR/Cas9 ribonucleoprotein (RNP) complex combined with the recombinant AAV serotype 6 (AAV6) donor vectors to introduce a gene of interest (GOI) fused with mCherry fluorescent reporter gene into the *AAVS1* safe harbor site. This approach leads to efficient transgene insertion and is applicable to precise genome editing of hiPSCs or other types of stem cells for research purposes.

## Introduction

Genome editing in hiPSCs offers a potential strategy for studying gene function and treating diseases. Unlike other gene-editing techniques, CRISPR/Cas9 is the most efficient, easy to perform, and amenable to multiplex gene editing [1, 2]. The CRISPR/Cas9 system consists of a Cas9 nuclease and a single guide RNA (sgRNA), which form a ribonucleoprotein (RNP) complex. Once the RNP complex binds to the target DNA, it generates double-stranded break (DSBs). The cells harness two endogenous DNA repair mechanisms, including the error-prone non-homologous end joining (NHEJ), which results in target gene disruption, and homology-directed repair (HDR), which results in precise genome editing [3–5]. The HDR-mediated gene correction or transgene insertion requires a DNA donor template in the form of a plasmid or single-stranded oligonucleotide (ssODN) containing left and right homology arms flanking the desired insertion.

In the past decade, CRISPR/Cas9-mediated gene editing has been utilized to correct genetic mutations in several disease-specific iPSCs, for example, thalassemia [6–8], hemophilia [9], primary hyperoxaluria type 1 (PH1) [10], and sickle cell disease [11]. Even though the ssODNs and plasmid vectors can introduce several base-pair mutations or transgene insertion, the gene targeting efficiency usually decreases with the larger transgene inserts [12, 13]. Recently, genome engineering using adeno-associated virus (AAV) vectors has emerged as a promising gene delivery tool due to its low immunogenicity and the ability to infect multiple human cell types in dividing and non-dividing cells [14]. A combination of CRISPR/Cas9 and AAV vectors provided an efficient knock-in of a DsRed reporter gene at the NRL locus in the human embryonic stem cells [15]. Furthermore, a highly efficient bi-allelic correction of sickle cell disease (SCD) mutation was reported using the Cas9 RNP complex combined with AAV6 transduction in a patient-derived iPSC line [16] and Townes-SCD mouse hematopoietic stem cells (HSCs) [17]. Notably, stable hemoglobin-A production was observed after autologous transplantation into Townes-SCD mice [17].

AAV is a single-stranded DNA virus comprising 4.7-kilobase (kb) genome in length. The AAV genome consists of *rep* (replication) and *cap* (capsid) genes flanked by two 145-bp inverted terminal repeats (ITRs) [18]. There are up to 12 serotypes of AAV vectors available [19, 20]. Previous studies demonstrated that AAV6 is the most efficient serotype for the transduction of primary human HSCs [21–24]. Recently, a successful genome editing in HSCs from sickle cell patients was achieved by using the AAV6 vectors [25]. Since the AAV is a replication-defective virus, it requires helper viruses such as adenovirus or herpes simplex virus for productive infection [26]. In the presence of the helper viruses, the AAVs can randomly integrate into the host chromosome. On the other hand, in the absence of the helper viruses, the AAVs preferably integrate into a specific site called *AAVS1* on human chromosome 19 [27]. For transgene knock-in, the transgenes are placed between the two ITRs in the AAV donor plasmid while the *rep*, *cap*, and helper genes are supplied in the helper plasmid. Production of recombinant helper-free AAV vectors requires co-transfection of the AAV donor and helper plasmids. Since the total size of the two ITRs is 290 bp and the homology arm size is 600 bp, the transgene size is limited to 3.8 kb for proper packaging efficiency [28].

In this protocol, we describe a step-by-step procedure to deliver the gene of interest (GOI) tagged with the mCherry reporter protein into the *AAVS1* safe harbor site in hiPSCs. Our protocol includes AAV6 vector production, purification, titration, nucleofection into hiPSCs, and clonal isolation. This approach could offer an efficient gene-editing platform for disease modeling and novel therapeutic strategies for genetic diseases.

## Materials and methods

The protocol described in this article is published on protocols.io, https://dx.doi.org/10.17504/protocols.io.yxmvmn2d9g3p/v3 and is included for printing as S1 File with this article.

### Results

**Fig 1:**
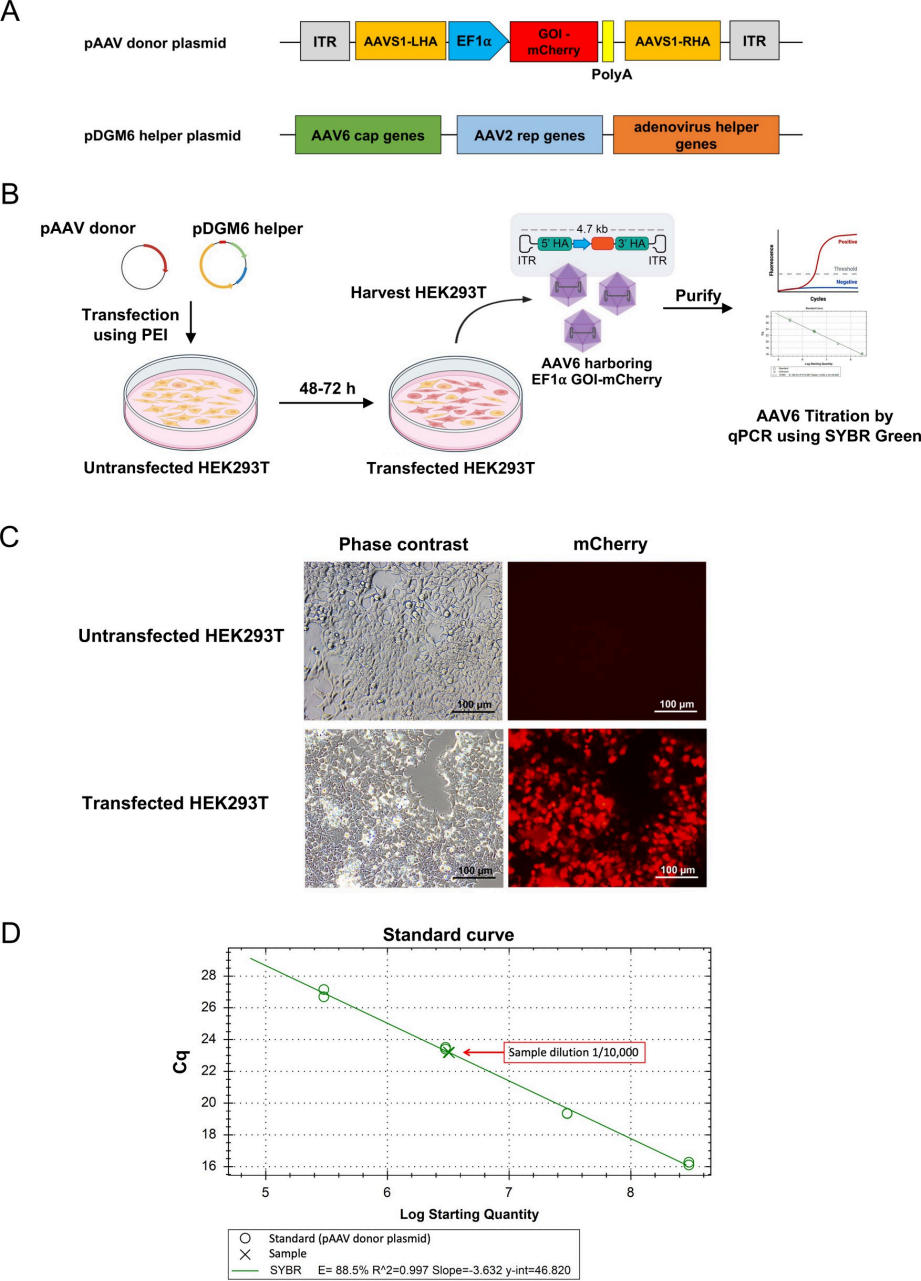
Production of AAV6 vectors in HEK293T cells. (A) The components of the pAAV donor plasmid and pDGM6 helper plasmid. The pAAV donor plasmid vector comprises ITR, left homology arm of *AAVS1* gene (AAVS1-LHA), EF1α promoter, the gene of interest (GOI) tagged with mCherry, polyA tail, right homology arm of *AAVS1* gene (AAVS1-RHA) and ITR. The pDGM6 helper plasmid consists of the AAV6 cap genes, the AAV2 rep genes, and the adenovirus helper genes. (B) Schematic of AAV6 production by co-transfection of the pAAV donor and pDGM6 helper plasmid vectors into HEK293T cells. (C) Fluorescence microscopic analysis of the untransfected and transfected HEK293T cells at 3 days post-transfection. Scale bar = 100 μm. (D) The qPCR standard curve was created by plotting the logarithmic DNA concentrations against Cq values.

In this protocol, we first transfected a pAAV donor plasmid and a pDGM6 helper plasmid (Fig 1A) into HEK293T cells for AAV6 production. The transfected HEK293T cells were harvested for isolation and purification of AAV6 (Fig 1B). Three days post-transfection, most of the transfected HEK293T cells expressed mCherry compared to the untransfected control (Fig 1C). We then harvested the AAV6 vectors from the HEK293T cells and purified them using the AAVpro^®^ Purification kit. The AAV6 titer was determined using primers specific to the ITR regions by quantitative PCR analysis. The standard curve was prepared by plotting the logarithmic DNA concentrations against the mean values of the quantification cycle (Cq). We obtained the correlation coefficient (R^2^) of the standard curve of 0.997 (slope −3.632) (Fig 1D). The sample data from dilution 1/10,000 was selected for calculating the AAV6 titer. From this experiment, we obtained the AAV6 titer of 6.36 × 10^9^ genome copies/μl.

**Fig 2:**
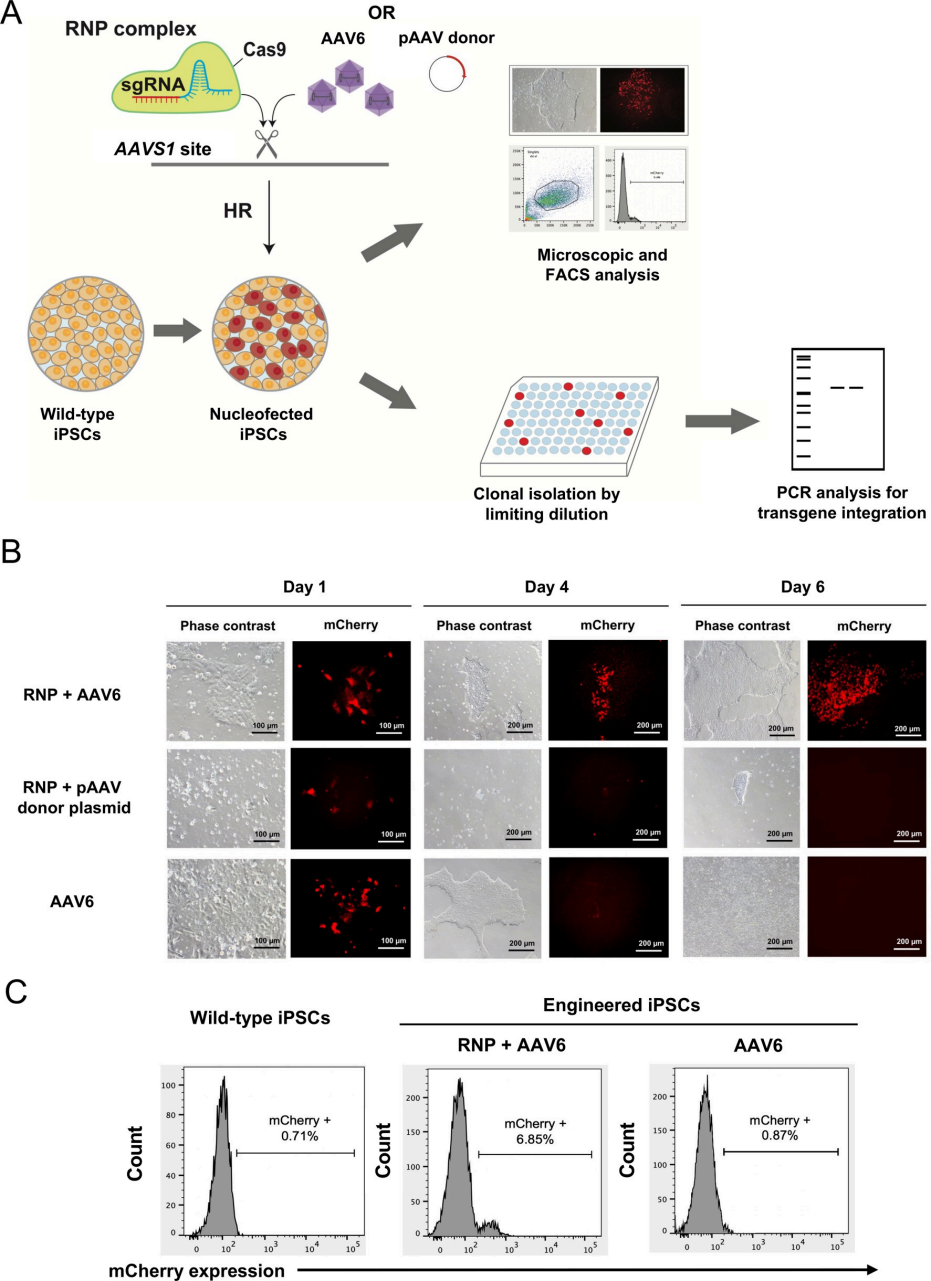
Nucleofection of human iPSCs. (A) Schematic diagram of the gene-editing strategy targeting the *AAVS1* locus using the CRISPR/Cas9 RNP complex and the recombinant AAV6 vectors in human iPSCs. HR = Homologous Recombination. (B) Microscopic fluorescence analysis of human iPSCs shows mCherry expression on days 1, 4 and day 6 post-nucleofection from three different conditions. Scale bar = 100 μm (Day 1) and 200 μm (Days 4 and 6). (C) The percentage of mCherry^+^ cells on day 6 post-nucleofection as analyzed by flow cytometry.

For knock-in of the GOI-mCherry gene into the *AAVS1* locus of the hiPSCs, we nucleofected the Cas9 RNP complex followed by adding the purified recombinant AAV6 vectors at the MOI of 100,000 (RNP + AAV6) (Fig 2A and 2B). For experimental controls, we delivered the transgene by nucleofecting the RNP complex with 0.5 μg of the pAAV donor plasmid (RNP + pAAV donor plasmid) or transducing the AAV6 vectors at the MOI of 100,000 alone (AAV6). On day 1 post-nucleofection, the mCherry^+^ cells were observed in all conditions. On days 4 and 6 post-nucleofection, most of the cells in the RNP + pAAV donor plasmid condition died while the cells in the RNP + AAV6 and AAV6 conditions survived and grew larger in size. However, the mCherry^+^ cells were observed only in the RNP + AAV6 condition. Flow cytometric analysis revealed that there were approximately 6.85% of mCherry^+^ cells under the RNP + AAV6 condition, while there were no mCherry^+^ cells under the AAV6 condition (Fig 2C). These results indicated that the combination of RNP complex and AAV6 vectors resulted in successful transgene delivery into human iPSCs. In contrast, the use of RNP complex with the donor plasmid resulted in poor transgene delivery and massive cell death while the transduction of AAV6 alone led to transient mCherry expression, which progressively diluted out during cell division.

**Fig 3:**
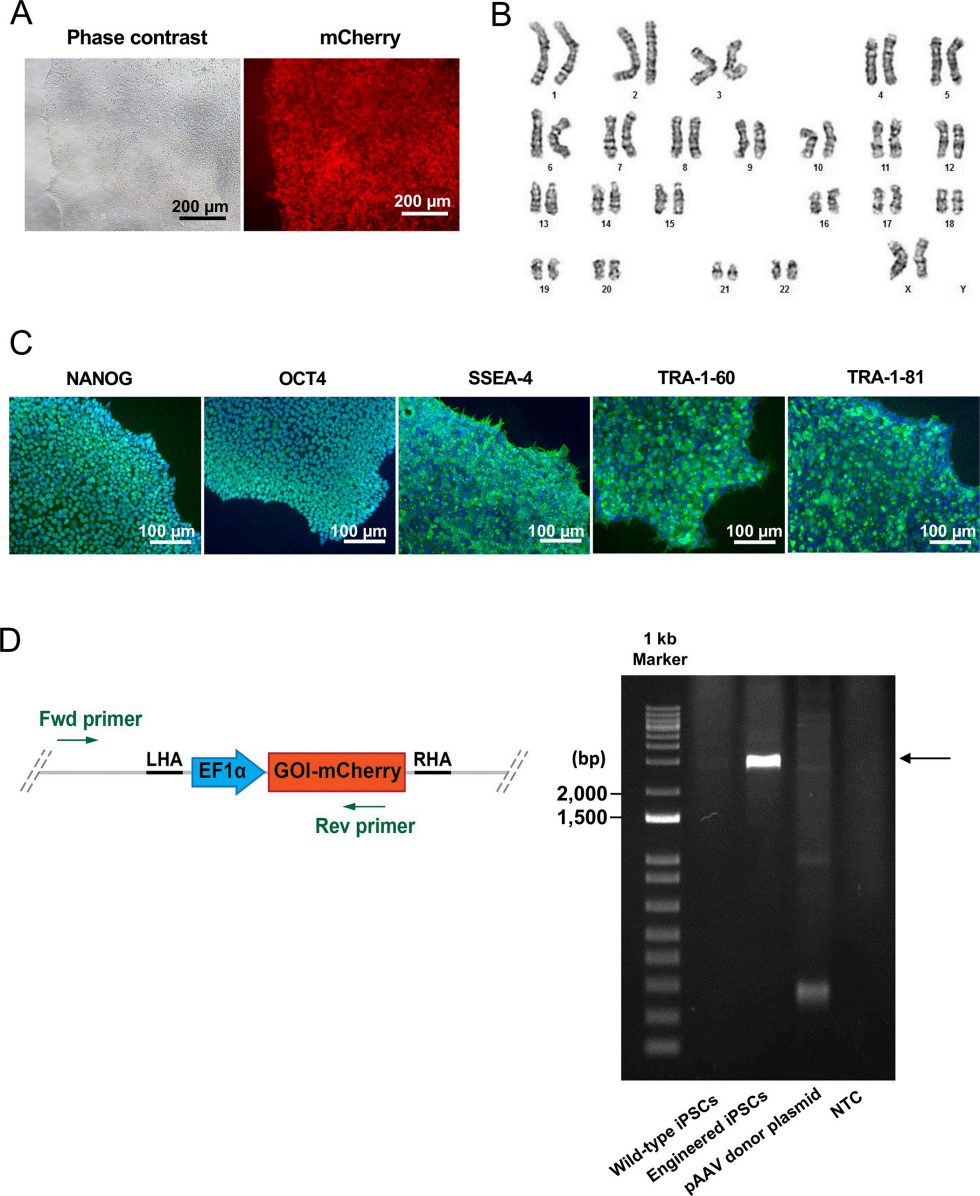
Clonal isolation and characterization of the genetically engineered human iPSCs. (A) The mCherry expression of human iPSCs after limiting dilution. Scale bar = 200 μm. (B) Karyotype analysis by standard G banding demonstrated that the engineered human iPSCs exhibited normal karyotype (46, XX). (C) Immunofluorescence staining of pluripotent markers NANOG, OCT4, SSEA-4, TRA-1-60 and TRA-1-81. The nuclei were stained with DAPI. Scale bar = 100 μm. (D) PCR amplification of genomic DNA extracted from the wild-type iPSCs, genetically-engineered iPSCs, pAAV donor plasmid and non-template control (NTC). The major band indicates a successful transgene knock-in at the *AAVS1* locus.

We next performed clonal isolation by limiting dilution and characterized the genetically engineered human iPSCs. After clonal isolation, the engineered iPSCs exhibited a homogeneous fluorescence distribution (Fig 3A). The engineered cells were expanded for karyotype analysis. The results showed that the cells exhibited normal karyotype (46, XX) (Fig 3B) and expressed pluripotent markers, including NANOG, OCT4, SSEA-4, TRA-1-60, and TRA-1-81 (Fig 3C). We performed PCR using the primers that amplify the upstream region of the *AAVS1* left-homology arm and the mCherry reporter protein as indicated by the green arrow (Fig 3D). The results demonstrated a successful transgene knock-in at the *AAVS1* safe harbor locus on human chromosome 19 (Fig 3D). Taken together, the CRISPR/Cas9 RNP complex in combination with the recombinant AAV6 vectors provide a precise gene delivery method for human iPSCs. The knowledge obtained from this study can be applied for transgene knock-in for both research and therapeutic applications.

## Supporting information

S1 File Step-by-step protocol, also available on protocol.io. (DOCX)

S2 File Uncropped gel of Fig 3D. (PDF)

## References

[1] Xue HY, Ji LJ, Gao AM, Liu P, He JD, Lu XJ. CRISPR-Cas9 for medical genetic screens: applications and future perspectives. J Med Genet. 2016;53(2):91–7. doi: 10.1136/jmedgenet-2015-103409 26673779

[2] Brookhouser N, Raman S, Potts C, Brafman DA. May I Cut in? Gene Editing Approaches in Human Induced Pluripotent Stem Cells. Cells. 2017;6(1). doi: 10.3390/cells6010005 28178187 PMC5371870

[3] Hockemeyer D, Soldner F, Beard C, Gao Q, Mitalipova M, DeKelver RC, et al. Efficient targeting of expressed and silent genes in human ESCs and iPSCs using zinc-finger nucleases. Nat Biotechnol. 2009;27(9):851–7. doi: 10.1038/nbt.1562 19680244 PMC4142824

[4] Mussolino C, Alzubi J, Fine EJ, Morbitzer R, Cradick TJ, Lahaye T, et al. TALENs facilitate targeted genome editing in human cells with high specificity and low cytotoxicity. Nucleic Acids Res. 2014;42(10):6762–73. doi: 10.1093/nar/gku305 24792154 PMC4041469

[5] Mali P, Yang L, Esvelt KM, Aach J, Guell M, DiCarlo JE, et al. RNA-guided human genome engineering via Cas9. Science. 2013;339(6121):823–6. doi: 10.1126/science.1232033 23287722 PMC3712628

[6] Xie F, Ye L, Chang JC, Beyer AI, Wang J, Muench MO, et al. Seamless gene correction of beta-thalassemia mutations in patient-specific iPSCs using CRISPR/Cas9 and piggyBac. Genome Res. 2014;24(9):1526–33. doi: 10.1101/gr.173427.114 25096406 PMC4158758

[7] Wattanapanitch M. Correction of Hemoglobin E/Beta-Thalassemia Patient-Derived iPSCs Using CRISPR/Cas9. Methods Mol Biol. 2211. 2020/12/19 ed2021. p. 193–211. 10.1007/978-1-0716-0943-9_14 33336279

[8] Wattanapanitch M, Damkham N, Potirat P, Trakarnsanga K, Janan M, Y UP, et al. One-step genetic correction of hemoglobin E/beta-thalassemia patient-derived iPSCs by the CRISPR/Cas9 system. Stem Cell Res Ther. 2018;9(1):46. doi: 10.1186/s13287-018-0779-3 29482624 PMC5828150

[9] Park CY, Kim DH, Son JS, Sung JJ, Lee J, Bae S, et al. Functional Correction of Large Factor VIII Gene Chromosomal Inversions in Hemophilia A Patient-Derived iPSCs Using CRISPR-Cas9. Cell Stem Cell. 2015;17(2):213–20. doi: 10.1016/j.stem.2015.07.001 26212079

[10] Esteve J, Blouin JM, Lalanne M, Azzi-Martin L, Dubus P, Bidet A, et al. Targeted gene therapy in human-induced pluripotent stem cells from a patient with primary hyperoxaluria type 1 using CRISPR/Cas9 technology. Biochem Biophys Res Commun. 2019;517(4):677–83. doi: 10.1016/j.bbrc.2019.07.109 31402115

[11] Huang X, Wang Y, Yan W, Smith C, Ye Z, Wang J, et al. Production of Gene-Corrected Adult Beta Globin Protein in Human Erythrocytes Differentiated from Patient iPSCs After Genome Editing of the Sickle Point Mutation. Stem Cells. 2015;33(5):1470–9. doi: 10.1002/stem.1969 25702619 PMC4628786

[12] Yang L, Guell M, Byrne S, Yang JL, De Los Angeles A, Mali P, et al. Optimization of scarless human stem cell genome editing. Nucleic Acids Res. 2013;41(19):9049–61. doi: 10.1093/nar/gkt555 23907390 PMC3799423

[13] Wang G, Yang L, Grishin D, Rios X, Ye LY, Hu Y, et al. Efficient, footprint-free human iPSC genome editing by consolidation of Cas9/CRISPR and piggyBac technologies. Nat Protoc. 2017;12(1):88–103. doi: 10.1038/nprot.2016.152 27929521 PMC5352979

[14] Kotterman MA, Schaffer DV. Engineering adeno-associated viruses for clinical gene therapy. Nat Rev Genet. 2014;15(7):445–51. doi: 10.1038/nrg3742 24840552 PMC4393649

[15] Ge X, Xi H, Yang F, Zhi X, Fu Y, Chen D, et al. CRISPR/Cas9-AAV Mediated Knock-in at NRL Locus in Human Embryonic Stem Cells. Mol Ther Nucleic Acids. 2016;5(11):e393. doi: 10.1038/mtna.2016.100 27898094 PMC5155318

[16] Martin RM, Ikeda K, Cromer MK, Uchida N, Nishimura T, Romano R, et al. Highly Efficient and Marker-free Genome Editing of Human Pluripotent Stem Cells by CRISPR-Cas9 RNP and AAV6 Donor-Mediated Homologous Recombination. Cell Stem Cell. 2019;24(5):821–8 e5. doi: 10.1016/j.stem.2019.04.001 31051134

[17] Wilkinson AC, Dever DP, Baik R, Camarena J, Hsu I, Charlesworth CT, et al. Cas9-AAV6 gene correction of beta-globin in autologous HSCs improves sickle cell disease erythropoiesis in mice. Nat Commun. 2021;12(1):686. doi: 10.1038/s41467-021-20909-x 33514718 PMC7846836

[18] Drouin LM, Agbandje-McKenna M. Adeno-associated virus structural biology as a tool in vector development. Future Virol. 2013;8(12):1183–99. doi: 10.2217/fvl.13.112 24533032 PMC3921901

[19] Gao G, Vandenberghe LH, Alvira MR, Lu Y, Calcedo R, Zhou X, et al. Clades of Adeno-associated viruses are widely disseminated in human tissues. J Virol. 2004;78(12):6381–8. doi: 10.1128/JVI.78.12.6381-6388.2004 15163731 PMC416542

[20] Gao G, Alvira MR, Somanathan S, Lu Y, Vandenberghe LH, Rux JJ, et al. Adeno-associated viruses undergo substantial evolution in primates during natural infections. Proc Natl Acad Sci U S A. 2003;100(10):6081–6. doi: 10.1073/pnas.0937739100 12716974 PMC156329

[21] Schuhmann NK, Pozzoli O, Sallach J, Huber A, Avitabile D, Perabo L, et al. Gene transfer into human cord blood-derived CD34(+) cells by adeno-associated viral vectors. Exp Hematol. 2010;38(9):707–17. doi: 10.1016/j.exphem.2010.04.016 20447441

[22] Veldwijk MR, Sellner L, Stiefelhagen M, Kleinschmidt JA, Laufs S, Topaly J, et al. Pseudotyped recombinant adeno-associated viral vectors mediate efficient gene transfer into primary human CD34(+) peripheral blood progenitor cells. Cytotherapy. 2010;12(1):107–12. doi: 10.3109/14653240903348293 19929455

[23] Song L, Kauss MA, Kopin E, Chandra M, Ul-Hasan T, Miller E, et al. Optimizing the transduction efficiency of capsid-modified AAV6 serotype vectors in primary human hematopoietic stem cells in vitro and in a xenograft mouse model in vivo. Cytotherapy. 2013;15(8):986–98. doi: 10.1016/j.jcyt.2013.04.003 23830234 PMC3711144

[24] Yang H, Qing K, Keeler GD, Yin L, Mietzsch M, Ling C, et al. Enhanced Transduction of Human Hematopoietic Stem Cells by AAV6 Vectors: Implications in Gene Therapy and Genome Editing. Mol Ther Nucleic Acids. 2020;20:451–8. doi: 10.1016/j.omtn.2020.03.009 32276210 PMC7150427

[25] Dever DP, Bak RO, Reinisch A, Camarena J, Washington G, Nicolas CE, et al. CRISPR/Cas9 beta-globin gene targeting in human haematopoietic stem cells. Nature. 2016;539(7629):384–9. doi: 10.1038/nature20134 27820943 PMC5898607

[26] Goncalves MA. Adeno-associated virus: from defective virus to effective vector. Virol J. 2005;2:43. doi: 10.1186/1743-422X-2-43 15877812 PMC1131931

[27] Kotin RM, Siniscalco M, Samulski RJ, Zhu XD, Hunter L, Laughlin CA, et al. Site-specific integration by adeno-associated virus. Proc Natl Acad Sci U S A. 1990;87(6):2211–5. doi: 10.1073/pnas.87.6.2211 2156265 PMC53656

[28] Grieger JC, Samulski RJ. Packaging capacity of adeno-associated virus serotypes: impact of larger genomes on infectivity and postentry steps. J Virol. 2005;79(15):9933–44. doi: 10.1128/JVI.79.15.9933-9944.2005 16014954 PMC1181570

