# Supplementary material for: A precise gene delivery approach for human induced pluripotent stem cells using Cas9 RNP complex and recombinant AAV6 donor vectors
Source: PLoS One. 2022 Jul 7;17(7):e0270963. doi: 10.1371/journal.pone.0270963 (PMC9262223; doi:10.1371/journal.pone.0270963)
Supplement: S1 File — (DOCX) [file pone.0270963.s001.docx]

**LAB PROTOCOL**

**A precise gene delivery approach for human induced pluripotent stem cells using Cas9 RNP complex and recombinant AAV6 vectors**

Koollawat Chupradit^1,2^, Nontaphat Thongsin^1,3^, Chatchai Tayapiwatana^2,4,5^, Methichit Wattanapanitch^1*^

^1^Siriraj Center for Regenerative Medicine, Research Department, Faculty of Medicine Siriraj Hospital, Mahidol University, Bangkok, Thailand

^2^ Center of Biomolecular Therapy and Diagnostic, Faculty of Associated Medical Sciences, Chiang Mai University, Chiang Mai, Thailand

^3^Department of Immunology, Faculty of Medicine Siriraj Hospital, Mahidol University, Bangkok, Thailand

^4^Division of Clinical Immunology, Department of Medical Technology, Faculty of Associated Medical Sciences, Chiang Mai University, Chiang Mai, Thailand

^5^Center of Innovative Immunodiagnostic Development, Faculty of Associated Medical Sciences, Chiang Mai University, Chiang Mai, Thailand

* Corresponding author

E-mail: [methichit.wat@mahidol.ac.th](mailto:methichit.wat@mahidol.ac.th) (MW)

**Materials**

**Production of AAV6 particles**

1. HEK293T cells
2. HEK293T culture medium: DMEM (Gibco^TM^ Cat. No. 12100046), 10% fetal bovine serum (FBS) (Sigma-Aldrich Cat. No. F7524), 2 mM GlutaMAX™ (Gibco™ Cat. No. 35050061), 1% penicillin/streptomycin (Gibco^TM^ Cat. No. 15140122)
3. Opti-MEM™ I Reduced Serum Medium (Gibco^TM^ Cat. No. 31985070)
4. DPBS (10X), no calcium, no magnesium (Invitrogen^TM^ Cat. No. 14200075)
5. UltraPure™ 0.5M EDTA, pH 8.0 (Invitrogen Cat. No. 15575020)
6. Trypsin-EDTA (0.25%), phenol red (Gibco^TM^ Cat. No. 25200072)
7. Purified pAAV donor and pDGM6 helper plasmid
8. Polyethylenimine (PEI) (Polysciences Cat. No. 23966-100)
9. Sodium butyrate (NaBu) (Sigma-Aldrich Cat. No. B5887-5G)
10. AAVpro^®^ Purification kit (All serotypes) (Takara Bio Cat. No. 6675)
11. Plasmid Midi kit (Qiagen Cat. No. 12145)
12. KAPA™ SYBR^®^ FAST qPCR Master Mix Kit (KAPABIOSYSTEMS Cat. No. KK4600)
13. QuickExtract™ DNA Extraction Solution (Lucigen Cat. No. QE0905T)
14. Primers targeting inverted terminal repeat (ITR) region
15. 150-mm tissue culture dishes
16. Multiplate^®^ PCR Plate™ 96-well
17. 25-cm^2^ and 75-cm^2^ tissue culture flasks
18. Hemacytometer
19. Sterile seropipettes, pipette tips
20. 15-ml and 50-ml conical tubes
21. Inverted microscope
22. Tabletop centrifuge
23. Quantitative PCR machine (CFX96 real-time, Bio-rad)
24. Tissue culture hood and humidified incubator (37°C, 5% CO_2_)

**Knock-in of GOI-mCherry in human iPSCs**

1. Human induced pluripotent stem cells (iPSCs)
2. Essential 8™ medium (Gibco^TM^ Cat. No. A1517001)
3. StemFlex™ medium (Gibco^TM^ Cat. No. A3349401)
4. DMEM/F-12, powder (Gibco^TM^ Cat. No. 12500062)
5. Matrigel^®^ (Corning^®^ Cat. No. 356234)
6. RevitaCell™ Supplement (Gibco^TM^ Cat. No. A2644501)
7. GeneArt™ Precision gRNA Synthesis Kit (Invitrogen^TM^ Cat. No. A29377)
8. P3 primary cell 4D-nucleofector X kit (Lonza Cat. No. V4XP-3032)
9. 20-µl Nucleocuvette™ Strip (supplied with the kit)
10. Alt-R^®^ S.p. Cas9 Nuclease V3 (Integrated DNA Technologies, IDT Cat. No. 1081058)
11. AAV6 particles
12. TrypLE™ Select Enzyme (Gibco^TM^ Cat. No. 12563011)
13. 24-well tissue culture plate
14. 15-ml conical tube
15. 1.5-ml Eppendorf tubes
16. Hemacytometer
17. 2% agarose gel: 2 g of agarose powder dissolved in 100 ml 1× TBE
18. Standard ladder 1 kb plus DNA ladder (Invitrogen^TM^ Cat. No. 10787018)
19. Novel juice (GeneDireX Cat. No. LD001-1000)
20. Gel chamber and power source
21. Gel documentation machine G:Box EF (SYNGENE)
22. NanoDrop 8000 (Thermo Fisher Scientific)
23. Thermal Cycler Model Tprofessional Basic Gradient 96 (Biometra)
24. Sterile seropipettes, pipette tips
25. Inverted fluorescence microscope
26. Tabletop centrifuge
27. Amaxa™ 4D-Nucleofector™ (Lonza)
28. Tissue culture hood and humidified incubator (37°C, 5% CO_2_)

**Validation of the GOI-mCherry expression by flow cytometry**

1. TrypLE™ Select Enzyme (Gibco)
2. Zombie Violet^TM^ Fixable viability dye (Biolegend Cat. No. 423113)
3. FAC buffer: 3% FBS in 1× PBS
4. 5-ml round-bottom tube
5. 1.5-ml Eppendorf tube
6. 15-ml conical tubes
7. Hemacytometer
8. 1% paraformaldehyde: 1 g of paraformaldehyde powder dissolved in 100 ml of 1× PBS
9. Sterile seropipettes, pipette tips
10. Flow cytometer (LSRFortessa™) (BD Biosciences)

**Clonal isolation by limiting dilution**

1. Essential 8™ medium (Gibco^TM^)
2. StemFlex™ medium (Gibco^TM^)
3. Matrigel^®^ (Corning^®^)
4. RevitaCell™ Supplement (Gibco^TM^)
5. 96-well flat-bottom plate
6. 1.5-ml Eppendorf tube
7. Hemacytometer
8. Multichannel pipette
9. Sterile seropipettes, pipette tips
10. Inverted fluorescence microscope
11. Tissue culture hood and humidified incubator (37°C, 5% CO_2_)

**Karyotyping**

1. Essential 8™ medium (Gibco^TM^)
2. Matrigel^®^ (Corning^®^)
3. 25-cm^2^ tissue culture flasks
4. Tissue culture hood and humidified incubator (37°C, 5% CO_2_)

**Characterization of iPSCs by immunofluorescence staining**

1. Anti-OCT4 Antibody, clone 9B7 (Sigma-Aldrich Cat. No. MABD76)
2. Anti-NANOG Antibody, clone 7F7.1 (Sigma-Aldrich Cat. No. MABD24)
3. Anti-Stage-Specific Embryonic Antigen-4 Antibody, clone MC-813-70 (Sigma-Aldrich Cat. No. MAB4304)
4. Anti-TRA-1-60 Antibody, clone TRA-1-60 (Sigma-Aldrich Cat. No. MAB4360)
5. Anti-TRA-1-81 Antibody, clone TRA-1-81 (Sigma-Aldrich Cat. No. MAB4381)
6. Goat anti-Mouse IgG (H+L) Cross-Adsorbed Secondary Antibody, Alexa Fluor™ 488 (Invitrogen Cat. No. A-11001)
7. Fluoromount-G™, with DAPI (Invitrogen Cat. No. 00-4959)
8. 24-well flat-bottom plate
9. 4% paraformaldehyde: 4 g of paraformaldehyde powder dissolved in 100 ml of 1× PBS
10. 3% Bovine serum albumin (BSA): 3 g of BSA were dissolved in 1× PBS (Merck Millipore)
11. 1% BSA: 1 g of BSA were dissolved in 1× PBS
12. 0.1% Triton™ X-100: 0.l ml of Triton™ X-100 were added in 100 ml of 1× PBS
13. 1.5-ml Eppendorf tube
14. Sterile seropipettes, pipette tip
15. Glass coverslip
16. Inverted fluorescence microscope

**Verification of GOI-mCherry in *AAVS1* site using PCR technique**

1. PureLink™ Genomic DNA Mini Kit (Invitrogen™ Cat. No. K182001)
2. NanoDrop 8000 (Thermo Fisher Scientific)
3. PCR tubes
4. PCR Thermal Cycler Model Tprofessional Basic Gradient 96 (Biometra)
5. 1% agarose gel: 1 g of agarose powder dissolved in 100 ml 1× TBE
6. Standard ladder 1 kb plus DNA ladder (Invitrogen^TM^ Cat. No. 10787018)
7. Novel Juice (GeneDireX Cat. No. LD001-1000)
8. Gel chamber and power source
9. Gel Documentation model G:Box EF (SYNGENE)

**Methods**

The following section describes the protocol to knock-in the gene of interest tagged with mCherry fluorescent protein (GOI-mCherry) into induced pluripotent stem cells (iPSCs) using the CRISPR/Cas9 system and adeno-associated virus serotype 6 (AAV6) donor vector. The protocol includes the production of AAV6 particles harboring GOI-mCherry, transfection of ribonucleoprotein (RNP) complex and transduction of AAV6 vector into iPSCs, validation of mCherry expression by flow cytometry, clonal isolation by limiting dilution and characterization of the engineered iPSCs for karyotyping, pluripotency and transgene integration.

1. **Production of the AAV6 particles harboring GOI-mCherry**

The AAV6 production is carried out by co-transfecting the pAAV donor plasmid and the pDGM6 helper plasmid into HEK293T cells. The donor plasmid contains inverted terminal repeat (ITR), left homology arm of the *AAVS1* gene (AAVS1-LHA), EF1α promoter, GOI-mCherry, polyA tail, right homology arm of the *AAVS1* gene (AAVS1-RHA), and ITR. The total size of the two ITRs is 290 bp while the size of each homology arm is 300 bp. The pDGM6 helper plasmid vector consists of the AAV6 cap genes, the AAV2 rep genes, and the adenovirus helper genes (Fig 1).

**
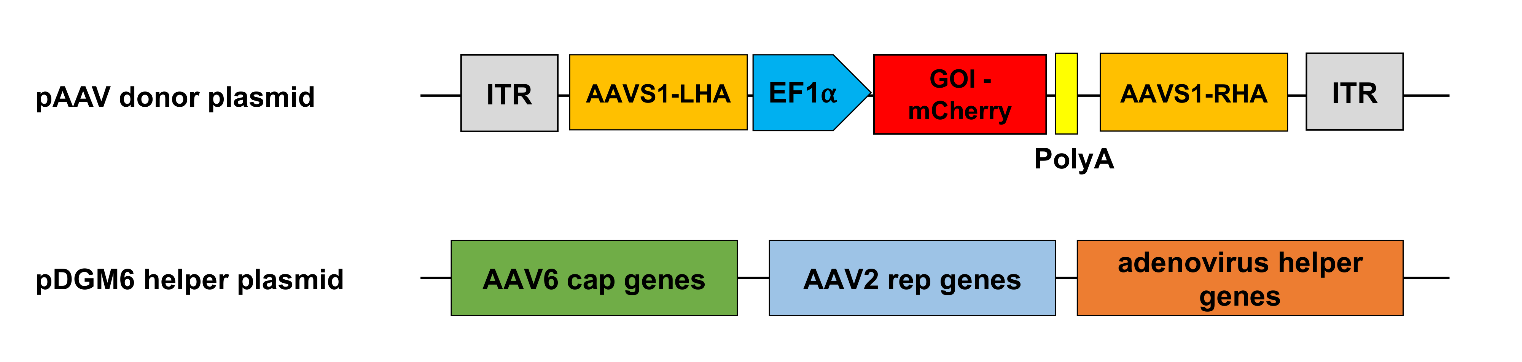
**

**Fig 1.** **Schematic diagram of pAAV donor plasmid and pDGM6 helper plasmid.**

1.1 Thawing of HEK293T cells

1. Quickly thaw HEK293T cells in a 37 ºC water bath and immediately transfer the cell suspension into a 15-ml conical tube containing 9 ml of DMEM basal medium.
2. Centrifuge the cells at 500 × g for 5 min.
3. Discard the supernatant and break the cell pellet by finger tapping.
4. Add 5 ml of HEK293T culture medium to the tube and gently pipette the cell suspension up and down.
5. Transfer the cell suspension to a 25-cm^2^ tissue culture flask.
6. Incubate the cells at 37 ºC, 5% CO_2_ for 3-4 days until the next passaging.

1.2 Passaging of HEK293T cells

1. Aspirate the culture medium and wash the cells with 1 × PBS.
2. Add 1 ml of 0.25% trypsin-EDTA and incubate the cells at 37 ºC, 5% CO_2_ for 5 min.
3. Add 5 ml of HEK293T culture medium, resuspend and transfer the cell suspension into a 15-ml conical tube.
4. Take out 10 µl of the cell suspension for counting using trypan blue exclusion assay to determine the cell number and viability.
5. Centrifuge the cell suspension at 500 × g for 5 min at room temperature.
6. After centrifugation, discard the supernatant and resuspend the cell pellet with 5 ml of HEK293T culture medium.
7. Transfer the cell suspension into a new 25-cm^2^ tissue culture flask at a seeding density of 2 - 5 × 10^4^ cells/cm^2^ and incubate at 37 ^o^C, 5% CO_2_.

1.3 Transfection of donor and helper plasmids into HEK293T cells

1. Passage HEK293T cells (Subheading 1.2, steps 1 - 6) and seed the cells at a seeding density of 3.3 × 10^6^ cells/75-cm^2^ flask for 3 flasks and incubate at 37^o^C, 5% CO_2_ for 3 - 4 days.
2. One day before transfection, perform HEK293T cell passaging (Subheading 1.2, step 1-6) and seed HEK293T cells onto two 150-mm culture dishes containing 20 ml of culture medium at a seeding density of 2.0 × 10^7^ cells/dish. Ensure even distribution of the cells in the plates.
3. Incubate the cells at 37^o^C, 5% CO_2_ overnight.

Note: The cells should be at least 80% confluency before transfection.

1. Prior to transfection, prepare the transfection mixture as follows.

a) Prepare DNA mixture by mixing 12 μg of the pAAV donor plasmid and 44 μg of the pDGM6 helper plasmid in 1 ml of opti-MEM^™^.

Note: The donor and helper plasmids should be prepared using a transfection-grade Plasmid Midi Kit. Since the pDGM6 helper plasmid is large (21,829 bp), we suggest increasing the culture volume for plasmid preparation. We use 400 ml of LB broth and harvest the culture after approximately 16–18 hours of growth for Midi-prep.

b) Prepare PEI mixture by diluting 224 μg of PEI with 1 ml of opti-MEM^™^.

1. Add the PEI mixture (step 4b) to the DNA mixture (step 4a). Vortex briefly and incubate for 15 min at room temperature.

Note: Do not add the DNA mixture to the PEI mixture.

1. Before the incubation time is over, add sodium butyrate (NaBu) solution to each HEK293T culture dish to a final concentration of 1 mM.
2. Add 1 ml of the transfection mix (step 5) into each HEK293T culture dish, mix thoroughly by swirling.
3. Incubate the cells at 37^o^C, 5% CO_2_ for 3 days.

1.4 Purification of AAV6 particles

In this section, the AAV6 particles are purified and concentrated using the AAVpro^®^ Purification kit (All Serotypes). Briefly, after co-transfection of the donor and helper plasmids into HEK293T cells, the AAV6 particles are produced inside the cells. The AAV6-producing HEK293T cells are harvested for purification of AAV6 particles following the AAVpro^®^ Purification Kit Midi (All Serotypes) product manual.

1. Dissociate the transfected HEK293T cells by adding 1/80 volume of 0.5 M EDTA (pH 8.0) to the culture medium and incubate at room temperature for 10 min.
2. After incubation, harvest the cells from the tissue culture dish by pipetting up and down.
3. Centrifuge the cells at 2,000 × g for 10 min at 4 °C and discard the culture supernatant.
4. Centrifuge the cells at 2,000 × g for 1 min and remove the supernatant completely.

Note: The remaining culture supernatant can interfere with the purification process.

1. Add 2 ml of AAV Extraction Solution A plus (provided by the kit) into the cells and vortex for 15 s.

Note: Ensure that there are no cell clumps.

1. Incubate the mixture at room temperature for 5 minutes and vortex again for 15 seconds.
2. Centrifuge at 4,000 - 9,000 × g for 10 min at 4 °C.
3. Transfer the supernatant to a new 15-ml conical tube and add 1/10 volume of AAV Extraction solution B.

Note: During this step, the sample can be stored at ˗80 °C and thawed in a 37 °C incubator before proceeding to the next step.

1. Transfer the sample to an Amicon Ultra-15, 100 kDa (provided by the kit), and follow the AAVpro® Purification Kit Midi (All Serotypes) product manual.
2. Aliquot the AAV6 particles into small volumes, store at –80 °C, and thaw the AAV6 particles at 4 °C before use.
   1. Quantification of AAV6 harboring GOI-mCherry

The AAV6 vector genome titer is determined by quantitative polymerase chain reaction (qPCR). We use the primers specific to the ITRs of the AAV6 vectors (1). Since the pAAV donor plasmid contains ITR regions, we use it for the preparation of standard control. The primer sequences are as follows.

Forward ITR primer: 5'-GGAACCCCTAGTGATGGAGTT-3'

Reverse ITR primer: 5'-CGGCCTCAGTGAGCGA-3'

1.5.1 Creating a standard curve using the pAAV donor plasmid

Prepare a 10-fold dilution series of the pAAV donor plasmids ranging from 3 × 10^8^ to 3 × 10^5^ copies. The copy numbers of the donor plasmids are calculated based on the following formula: <https://tools.thermofisher.com/content/sfs/brochures/cms_042486.pdf>

Here is an example of data calculation.

1. Calculate the mass of a single plasmid molecule using the following formula.

$$n=\frac{(m \times N_{A})}{M}$$

where n is the number of base pairs, m is the mass of DNA, N_A_ is Avogadro’s number (6.02 × 10^23^ bp/mole), and M is the average molecular weight of a double-stranded DNA (660 g/mole).

$m=\left[ n \right][1.096e˗21\frac{g}{\mathrm{bp}}$]

In this case, the plasmid size is 5,933 bp, and the plasmid concentration is 1.43 µg/µl. Thus, the mass of a single plasmid is 6.502 × 10^˗18^ g.

1. Calculate the mass of the donor plasmids ranging from 3 × 10^8^ to 3 × 10^5^ copies.

The following table represents the calculated plasmid mass to obtain the copy numbers.

| **Copy number** | **Mass of a single plasmid** | **Mass of plasmid DNA (g)** |
| --- | --- | --- |
| 300,000,000 | × 6.502 × 10^˗18^ g | 1.9506 ×10^˗09^ |
| 30,000,000 |  | 1.9506 × 10^˗10^ |
| 3,000,000 |  | 1.9506 × 10^˗11^ |
| 300,000 |  | 1.9506 × 10^˗12^ |

1. Calculate the concentrations of the donor plasmid needed when pipetting 5 µl of the donor plasmid DNA into the PCR reaction.

| **Copy number** | **Mass of plasmid DNA needed (g)** | ÷ 5 µl | **Final concentration of plasmid DNA (g/µl)** |
| --- | --- | --- | --- |
| 300,000,000 | 1.9506 × 10^˗09^ |  | 3.90154 × 10^˗10^ |
| 30,000,000 | 1.9506 × 10^˗10^ |  | 3.90154 × 10^˗11^ |
| 3,000,000 | 1.9506 × 10^˗11^ |  | 3.90154 × 10^˗12^ |
| 300,000 | 1.9506 × 10^˗12^ |  | 3.90154 × 10^˗13^ |

1. Prepare serial dilutions using the formula C_1_V_1_ = C_2_V_2_ (in duplicate).

| **Dilution** | **Initial concentration (g/µl) (C_1_)** | **Volume of plasmid DNA (µl)**  **(V_1_)** | **Volume of diluent (µl)** | **Final volume (µl)**  **(V_2_)** | **Final concentration (g/µl)**  **(C_2_)** | **Results of the copies per 5 µl** |
| --- | --- | --- | --- | --- | --- | --- |
| 1 | 1.43× 10^˗06^ | 5 | 495 | 500 | 1.43× 10^˗08^ |  |
| 2 | 1.43× 10^˗08^ | 10 | 90 | 100 | 1.43× 10^˗09^ |  |
| 3 | 1.43× 10^˗09^ | 27.34 | 72.66 | 100 | 3.90× 10^˗10^ | 300,000,000 |
| 4 | 3.90× 10^˗10^ | 10 | 90 | 100 | 3.90× 10^˗11^ | 30,000,000 |
| 5 | 3.90× 10^˗11^ | 10 | 90 | 100 | 3.90× 10^˗12^ | 3,000,000 |
| 6 | 3.90× 10^˗12^ | 10 | 90 | 100 | 3.90× 10^˗13^ | 300,000 |

1.5.2 Sample preparation

The AAV6 genome is extracted from the purified AAV6 particles using QuickExtract™ DNA Extraction solution. Alternatively, the AAV6 vector genome can also be extracted using the AAVpro^®^ Titration Kit (for Real-Time PCR) (Takara Bio).

1. Add 10 μl of the purified AAV6 product (heading 1.4) to 90 μl of QuickExtract™ DNA Extraction solution, vortex continuously for 15 s.
2. Incubate at 65 ^o^C for 10 min.
3. After incubation, vortex for 15 s.
4. Heat the sample at 98 ^o^C for 10 min to stop the enzymatic reaction.
5. The sample can be used immediately or stored at -20 ^o^C for up to 1 week.
6. Dilute the AAV6 genome according to the dilution scheme in the table below.

| **Dilution** | **Volume of the sample (µl)** | **Volume of water (µl)** | **Dilution factor** | **Total dilution factor** |
| --- | --- | --- | --- | --- |
| Viral genome (step 5) |  |  | 10 | 10 |
| 1 | 5 | 45 | 10 | 100 |
| 2 | 5 | 495 | 100 | 10,000 |
| 3 | 5 | 45 | 10 | 100,000 |
| 4 | 5 | 45 | 10 | 1,000,000 |
| 5 | 5 | 45 | 10 | 10,000,000 |
| 6 | 5 | 45 | 10 | 100,000,000 |

1.5.3 Quantitative PCR (qPCR) analysis

1. Prepare qPCR reaction for the quantification of AAV6 vectors following the table below.

| **Components** | **1 reaction (µl)** |
| --- | --- |
| KAPA™ SYBR^®^ FAST qPCR Master Mix | 10 |
| 100 μM Forward primer | 0.15 |
| 100 μM Reverse primer | 0.15 |
| Nuclease free water | 4.7 |
| Total volume | 15 |

1. Aliquot 15 µl of SYBR^®^ master mix into each well of multiplate^®^ PCR Plate™ 96-well.
2. Add 5 µl of the diluted pAAV donor plasmid (subheading 1.5.1, step 4) or the diluted AAV6 genome (subheading 1.5.2, step 5) to multiplate^®^ PCR Plate™ 96-well containing the SYBR^®^ master mix (step 2).

Note: Prepare the reaction mix in duplicate and include a non-template control (NTC) by omitting any DNA template for monitoring contamination or primer-dimer formation.

1. Spin down the plate to bring the sample to the bottom of the plate.
2. Place the multiplate^®^ PCR Plate™ 96-well plate in qPCR machine (CFX96 real-time, Bio-rad) and run the following program.

| **Step** | **Temperature** | **Duration** | **Cycle** | **Signal acquisition** |
| --- | --- | --- | --- | --- |
| Initial denaturation | 98 °C | 3 min | 1 |  |
| Denaturation | 98 °C | 15 sec | 40 |  |
| Annealing/extension | 58 °C | 30 sec |  | Plate Read |
| Malting curve analysis | 65-95 °C | 10 min |  | Plate Read |

1.5.4 Calculation of AAV6 titers

1. Perform data analysis using the CFX Manager™ software to obtain Cq values.
2. Plot standard curve using plasmid vector copies of 3 ⨉ 10^5^ - 3 ⨉ 10^8^.
3. Obtain Cq values of samples and calculate the titer using a linear equation

y = mx + c

where, y = y values (Cq)

m = slope

x = x values (AAV6 particles)

c = the intercept on the y-axis (y-int)

**
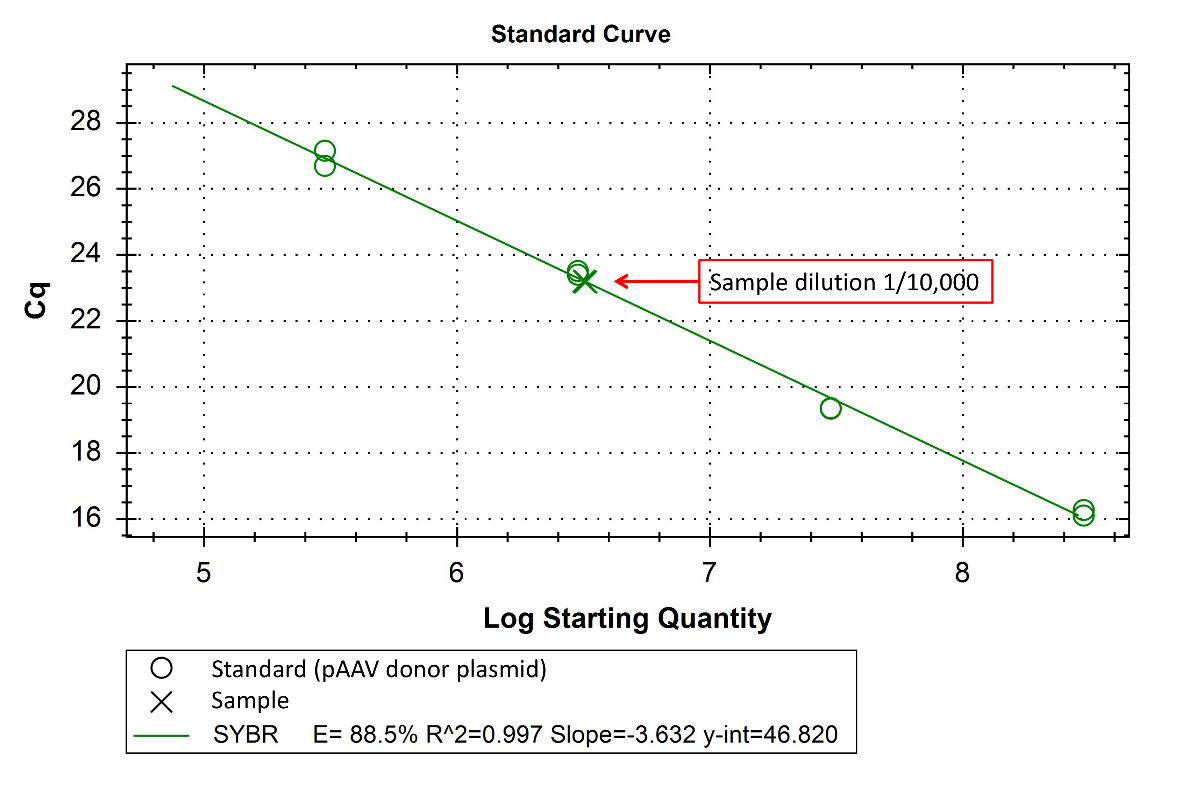
**

1.5.5 Sample data calculation

Determine the AAV6 titer using Cq from samples within the standard curve. In this example, the unknown sample represents the dilution of 1/10,000.

Using the formula y = -3.632x + 46.82, we obtain AAV6 titers as shown in the table.

|  | Replicate 1 | Replicate 2 |
| --- | --- | --- |
| y (Cq values) | 23.21 | 23.19 |
| x (viral particles of dilution 1/10,000) | 3.16 × 10^6^ | 3.20 × 10^6^ |
| Viral particles in 5 µl | 3.16 × 10^10^ | 3.20 × 10^10^ |
| Titer (viral particles/µl) | 6.32 ×10^9^ | 6.41 ×10^9^ |

Therefore, the average AAV6 titer is 6.36 × 10^9^ viral particles/µl.

1. **Knock-in of GOI-mCherry in human iPSCs**

Transgene knock-in can be achieved by delivering the RNP complex consisting of Cas9 protein and the *in vitro* transcribed (IVT) single guide RNA (sgRNA) into human iPSCs followed by transducing the purified recombinant AAV6 vectors. This section describes the procedure from the generation of the IVT sgRNA, transfection of the RNP complex, and transduction of the AAV6 vector.

2.1 Generation of sgRNA using *in vitro* transcription (IVT)

We use the sgRNA targeting the human *AAVS1* locus on chromosome 19 according to the previously published sequence (2). The gRNA DNA template assembly and the generation of IVT sgRNA are performed according to the GeneArt™ Precision gRNA Synthesis Kit’s instruction.

1. Order the primers for gRNA DNA template assembly as follows:

Fwd_AAVS1 5’- TAATACGACTCACTATAGGGGGCCACTAGGGACAG -3’

Rev_AAVS1 5’- TTCTAGCTCTAAAACATCCTGTCCCTAGTGGCCCC -3’

1. Perform the PCR assembly to generate the gRNA DNA template using the universal forward and reverse primers provided in the kit and the primer pair (step 1).
2. Generate the sgRNA by *in vitro* transcription using the gRNA DNA template and the TranscriptAid reaction mix (provided in the kit).
3. Determine the quality of the IVT sgRNA by gel electrophoresis using 2% agarose gel.

Note: The expected band is around 100 bp.

1. Purify the IVT sgRNA using the GeneJet^TM^ RNA purification Micro Columns. Elute the sgRNA using 10 µl of nuclease-free water.

Note: The elution volume depends on the desired sgRNA concentration. However, using elution volume less than 10 µl may reduce the sgRNA yield.

1. Determine the purified sgRNA concentration using Nanodrop.

Note: The expected concentration of the purified sgRNA is approximately 2 – 4 µg/µl.

1. The sgRNA can be used immediately or stored at -20 ^°^C until use. For prolonged storage (> 1 month), it is recommended to store the sgRNA at -80 ^°^C.

2.2 Transfection of the RNP complex and transduction of the AAV6 vector into iPSCs

Culture of iPSCs can be performed according to the previously published protocol (3). The MUSIi011-A is an iPSC line that was reprogrammed from peripheral blood T lymphocytes of a healthy female donor established in our laboratory (4). For knock-in of GOI-mCherry, we culture the iPSCs in a well of 12-well plate until 70% confluence before nucleofection.

1. Pre-treat the iPSCs with Essential 8^TM^ medium supplemented with RevitaCell^TM^ and incubate at 37 °C for 2 h.
2. Prepare a well of 24-well plate by coating with the 1:40 diluted Matrigel^®^ for an hour at room temperature.
3. Replace the diluted Matrigel^®^ with 0.4 ml of StemFlex^TM^ medium supplemented with RevitaCell^TM^ and incubate the plate at 37 °C, 5% CO_2_.
4. After 2-hour incubation, wash the cells (step 1) with 1 × PBS twice.
5. Add 0.5 ml TrypLE^TM^ Select and incubate at 37 °C for 6 min.
6. Following incubation, dissociate the cells by pipetting up and down.
7. Transfer single cells into a 15-ml conical tube. Rinse the well with 1 ml DMEM/F-12 and transfer the cell suspension to the same tube.
8. Centrifuge the cells at 500 × g for 5 min at room temperature.
9. Discard the supernatant and be careful not to disturb the cell pellet.
10. Resuspend the cells with 1 ml of Essential 8^TM^ medium, take out 10 µl of cell suspension for counting.
11. For each reaction, transfer 5 × 10^5^ cells into a 1.5-ml Eppendorf tube and centrifuge the cells at 500 × g for 5 min at room temperature.
12. During centrifugation, prepare the RNP complex by mixing 150 µg/ml Alt-R^®^ S.p. Cas9 Nuclease V3 with 90 µg/ml sgRNA, and incubate at room temperature for 10 min.

Note: We mix Cas9 protein and sgRNA at 1:3 molar ratio. Mole of sgRNA is calculated by NEBioCalculator^TM^ v1.10.0 using ssRNA: Mass to Moles Converter mode.

1. Prepare P3 nucleofection^TM^ solution by mixing 16.4 µl of Nucleofector^TM^ solution and 3.6 µl of supplement. Mix thoroughly by pipetting and keep the mixture on ice.
2. Use the premixed P3 nucleofector solution to resuspend the cell pellet (step 11).
3. Add the RNP complex to the cell suspension and gently mix by pipetting up and down.

Note: For the RNP and pAAV donor plasmid condition, add 0.5 µg of donor plasmid to the reaction.

1. Carefully transfer the mixture into a well of Nucleocuvette^TM^ Strip without introducing any air bubbles. Ensure that the cell/RNP mix is deposited at the bottom of the well.

Note: Do not leave the cells in the nucleofector solution for too long since it decreases the transfection efficiency.

1. Close the lid and place the Nucleocuvette^TM^ Strip into the retainer of the Amaxa™ 4D-Nucleofector™ X Unit.
2. Perform nucleofection using the CA-137 program.
3. After nucleofection, resuspend the cells with 80 µl of prewarmed StemFlex^TM^ medium supplemented with RevitaCell^TM^.
4. Transfer 100 µl of the cell suspension into the prepared well (step 3).
5. Add the purified AAV6 vectors (subheading 1.4) at an MOI of 100,000.

Note: For transduction of 500,000 iPSCs, add 7.86 µl of the purified AAV6 vectors according to the calculated viral titer of 6.36 × 10^9^ viral particles/µl. Incubate at 37 °C, 5% CO_2_.

1. Change the medium daily, RevitaCell^TM^ should be supplemented for at least 2 days to enhance the cell viability after transduction.
2. Culture the transduced iPSCs until confluent.
3. **Validation of the GOI-mCherry expression by flow cytometry**

The mCherry expression can be observed using an inverted fluorescence microscope. To determine the knock-in efficiency, the transduced iPSCs are dissociated and harvested for flow cytometric analysis.

1. Aspirate the medium and wash the cells with 1 ml of sterile 1 × PBS.
2. Add 0.5 ml of TrypLE^TM^ Select to dissociate the cells. Incubate at 37 °C for 6 min.
3. Transfer the cell suspension to a 15-ml conical tube.
4. Wash the well with DMEM/F-12 twice and transfer the cell suspension to the same tube.
5. Take out 10 µl of cell suspension for counting with a hemacytometer.
6. Transfer 10^6^ cells into a 1.5-ml tube and add 1 ml of FACS buffer.
7. Centrifuge at 500 × g for 5 min at room temperature.
8. During centrifugation, dilute the Zombie Violet^TM^ Fixable viability dye at 1:500 dilution in FACS buffer.
9. Discard the supernatant and resuspend the cells in 100 µl of the diluted Zombie Violet solution.
10. Incubate the cells for 15 min in the dark at room temperature.
11. Add 1 ml of FACS buffer and centrifuge at 500 × g for 5 min at room temperature.
12. Discard the supernatant and resuspend the cell pellet with 350 µl of 1% paraformaldehyde and transfer the cell suspension to a 5-ml round-bottom tube.
13. Measure the fluorescence signals using the appropriate lasers and filters using a Becton Dickinson FACS LSRFortessa model (BD Biosciences).


1. **Clonal isolation by limiting dilution**

The mCherry^+^ clones can be isolated by limiting dilution. The number of plates can be adjusted accordingly depending on the transduction efficiency.

1. Pre-coat a 96-well flat-bottom plate with 50 µl of 1:40-diluted Matrigel^®^ and incubate at 37 °C, 5% CO_2_ for 1 hour.
2. Aspirate the medium from the transduced iPSCs and replace with Essential™ 8 medium supplemented with RevitaCell^TM^ and incubate at 37 °C, 5% CO_2_ for 2 hours.
3. After incubation, aspirate the medium and wash the cells with 1 × PBS.
4. Add TrypLE^TM^ Select to dissociate the cells. Incubate at 37 °C for 6 min.
5. Transfer the cell suspension to a 15-ml conical tube.
6. Wash the well with DMEM/F-12 twice and transfer the cell suspension into the same tube.
7. Take out 10 µl of the cell suspension for counting.
8. Centrifuge the cells at 500 × g for 5 min at room temperature.
9. Aspirate the medium, resuspend the cells in StemFlex™ Medium supplemented with RevitaCell^TM^ to obtain the seeding density of 4 cells/100 µl/well.
10. Aspirate the diluted Matrigel^®^ (step 1) and add 100 µl of the cell suspension to each well of the coated 96-well flat-bottom plate. Incubate the cells at 37^o^C, 5% CO_2_.
11. Change the medium every other day. RevitaCell^TM^ should be supplemented for at least two days to enhance the cell viability.
12. Mark the wells that contain a single colony. When the single colony grows, subculture the mCherry^+^ positive colony to a well of 24-well tissue culture plate for expansion.
13. **Karyotyping**

The engineered iPSCs is determined the genomic integrity after clonal isolation by karyotyping using a standard G-banding technique.

1. Culture the iPSCs in a 25-cm2 tissue culture flask until confluent.

2. Send the iPSCs to the Department of Obstetrics and Gynecology, Faculty of Medicine Siriraj Hospital, Mahidol University to analyze 25 metaphases at the resolution of 450-500 by the standard Giemsa banding protocol.

1. **Characterization of the engineered iPSCs by immunofluorescence staining**

The isolated mCherry^+^ clone is analyzed for the expression of pluripotent markers including NANOG, OCT4, SSEA-4, TRA-1-60, and TRA-1-81.

1. Seed the iPSCs in a 24-well flat-bottom plate and incubate at 37 °C, 5% CO_2_ for 3-4 days. Change the medium daily.
2. Aspirate the culture medium and wash the cells with 1 × PBS.
3. Fix the cells with 300 µl of 4% paraformaldehyde in PBS and incubate at room temperature for 20 min.
4. After incubation, wash the cells with 1 × PBS twice (5 min each).
5. For NANOG and OCT4 (intracellular staining), add 300 µl of 0.1% Triton X-100 in PBS to permeabilize the cells and incubate for 10 min at room temperature.
6. After incubation, wash the cells gently with 1 × PBS three times (5 min each).
7. Add 300 µl of 3% BSA in PBS to block non-specific binding of the antibodies and incubate for 1 h at room temperature.
8. Prepare proper amounts of primary antibodies in 1% BSA in PBS.
9. Add 250 µl of diluted primary antibodies to each well. Incubate at 4 °C overnight.
10. After incubation, wash the cells with 1 × PBS for three times (5 min each).
11. Prepare proper amount of the secondary antibody, AlexaFluor488 FITC, in 1% BSA in PBS.
12. Add 250 µl of the diluted secondary antibody to the wells. Incubate for 1 h in the dark at room temperature.
13. Wash the well with 1 × PBS for three times (5 min each).
14. Add 25 µl of Fluoromount-G™ with DAPI to the well and apply a glass coverslip to each well.
15. Observe fluorescent signals under a fluorescence microscope.

| **Antibody** | **Dilution** |
| --- | --- |
| NANOG | 1: 100 |
| OCT4 | 1: 500 |
| SSEA-4 | 1: 100 |
| TRA-1-60 | 1: 100 |
| TRA-1-81 | 1: 100 |
| Goat anti-Mouse Alexa Fluor™ 488 | 1: 500 |

1. **Verification of GOI-mCherry integration at the *AAVS1* site using PCR technique**

To verify whether GOI-mCherry is successfully integrated into the *AAVS1* safe harbor locus, the genomic DNA of the wild-type iPSCs and the engineered iPSCs are extracted for PCR amplification using specific primers.

Forward primer: 5'- TCTTGTAGGCCTGCATCATCACC -3'

Reverse mCherry primer: 5'- TCGTAGGGGCGGCCCTCGC -3'

1. Extract genomic DNA from 1 × 10^6^ wild-type iPSCs, engineered iPSCs using PureLink™ Genomic DNA Mini Kit and determine the genomic DNA concentration by NanoDrop.
2. Prepare the PCR reaction following the table below.

| **Component** | **1 reaction (µl)** |
| --- | --- |
| DreamTaq Green PCR Master Mix (2X) | 10 |
| 10 μM Forward | 0.2 |
| 10 μM Reverse mCherry | 0.2 |
| Nuclease-free water | 8.6 |
| Total volume | 19 |

1. Add 1 µl of the genomic DNA template or pAAV donor plasmid (step 1) into the PCR reaction.

Note: Prepare the reaction mix, including a non-template control (NTC) as a negative control.

1. Spin down the PCR tube to bring the sample to the bottom of the tube.
2. Place the PCR tube in a PCR Thermal Cycler, Model Tprofessional Basic Gradient 96 (Biometra), and run the following program.

| **Step** | **Temperature** | **Duration** | **Cycle** |
| --- | --- | --- | --- |
| Initial denaturation | 95 °C | 2 min |  |
| Denaturation | 95 °C | 20 sec | 35 |
| Annealing | 70 °C | 30 sec |  |
| Extension | 72 °C | 2 min 10 sec |  |
| Final extension | 72 °C | 10 min |  |

1. Take 5 µl of the PCR product and mix with 1 µl Novel Juice.
2. Perform DNA agarose gel electrophoresis and visualize DNA bands upon UV illumination using Gel Documentation model G:Box EF (SYNGENE).

**References**

1. Aurnhammer C, Haase M, Muether N, Hausl M, Rauschhuber C, Huber I, et al. Universal real-time PCR for the detection and quantification of adeno-associated virus serotype 2-derived inverted terminal repeat sequences. Hum Gene Ther Methods. 2012;23(1):18-28.

2. Lyu C, Shen J, Wang R, Gu H, Zhang J, Xue F, et al. Targeted genome engineering in human induced pluripotent stem cells from patients with hemophilia B using the CRISPR-Cas9 system. Stem Cell Res Ther. 2018;9(1):92.

3. Thongsin N, Wattanapanitch M. Generation of B2M bi-allelic knockout human induced pluripotent stem cells (MUSIi001-A-1) using a CRISPR/Cas9 system. Stem Cell Res. 2021;56:102551.

4. Netsrithong R, Promnakhon N, Boonkaew B, Vatanashevanopakorn C, Pattanapanyasat K, Wattanapanitch M. Generation of two induced pluripotent stem cell lines (MUSIi011-A and MUSIi011-B) from peripheral blood T lymphocytes of a healthy individual. Stem Cell Res. 2019;39:101487.
