# Supplementary material for: A precise gene delivery approach for human induced pluripotent stem cells using Cas9 RNP complex and recombinant AAV6 donor vectors
Source: PLoS One. 2022 Jul 7;17(7):e0270963. doi: 10.1371/journal.pone.0270963 (PMC9262223; doi:10.1371/journal.pone.0270963)
Supplement: S2 File — (PDF) [file pone.0270963.s002.pdf]

S2 file

A precise gene delivery approach for human induced pluripotent stem cells using Cas9 RNP complex and recombinant AAV6 donor vectors

Koollawat Chupradit, Nontaphat Thongsin, Chatchai Tayapiwatana, Methichit Wattanapanitch*

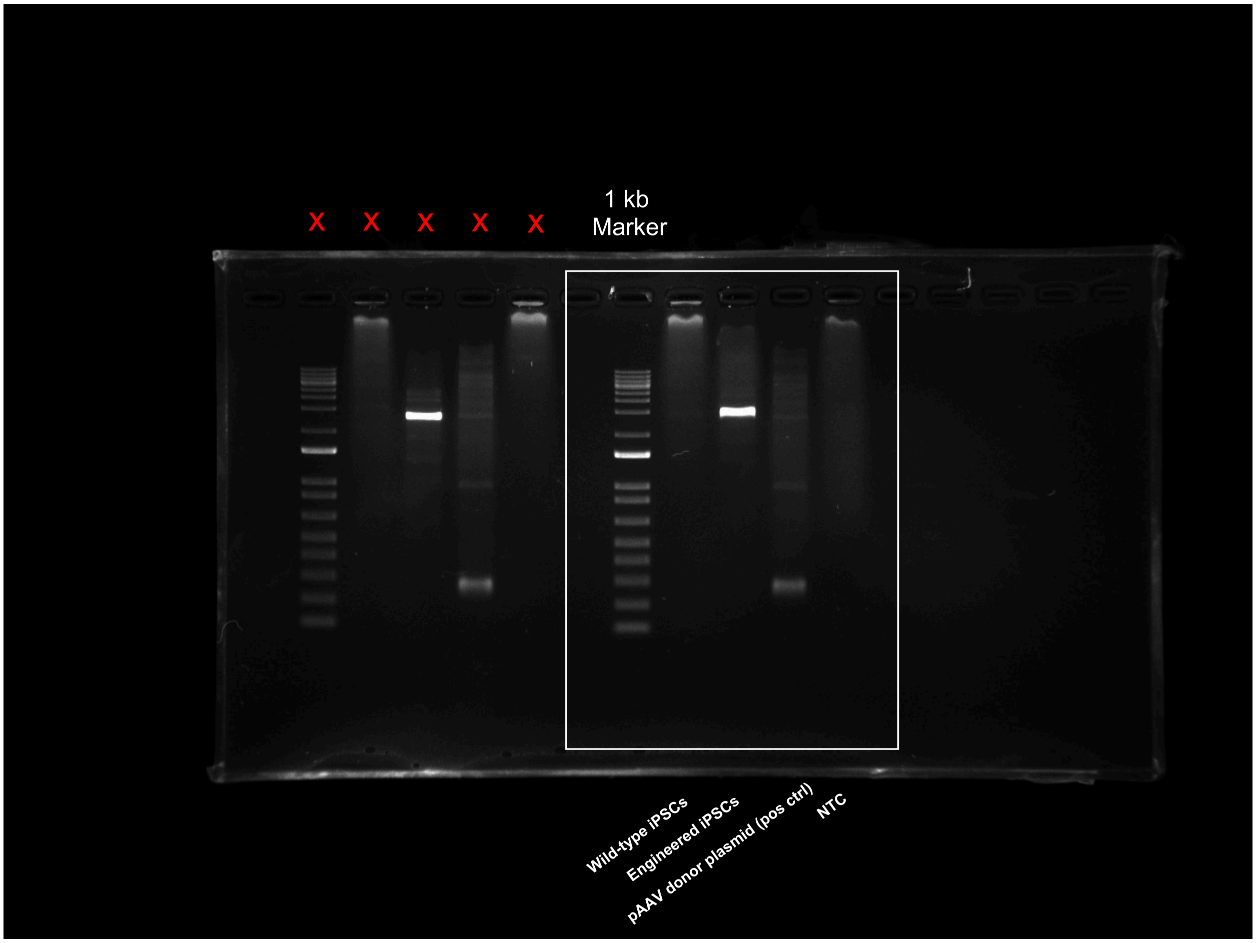

Uncropped gel of Figure 3D depicting cropped area (white line)
